# Supplementary material for: SARS-CoV-2 infection induces sustained humoral immune responses in convalescent patients following symptomatic COVID-19
Source: Nat Commun. 2021 Mar 22;12:1813. doi: 10.1038/s41467-021-22034-1 (PMC7985370; doi:10.1038/s41467-021-22034-1)
Supplement: Supplementary file 1 — Supplementary Information [file 41467_2021_22034_MOESM1_ESM.pdf]

Supplementary Information

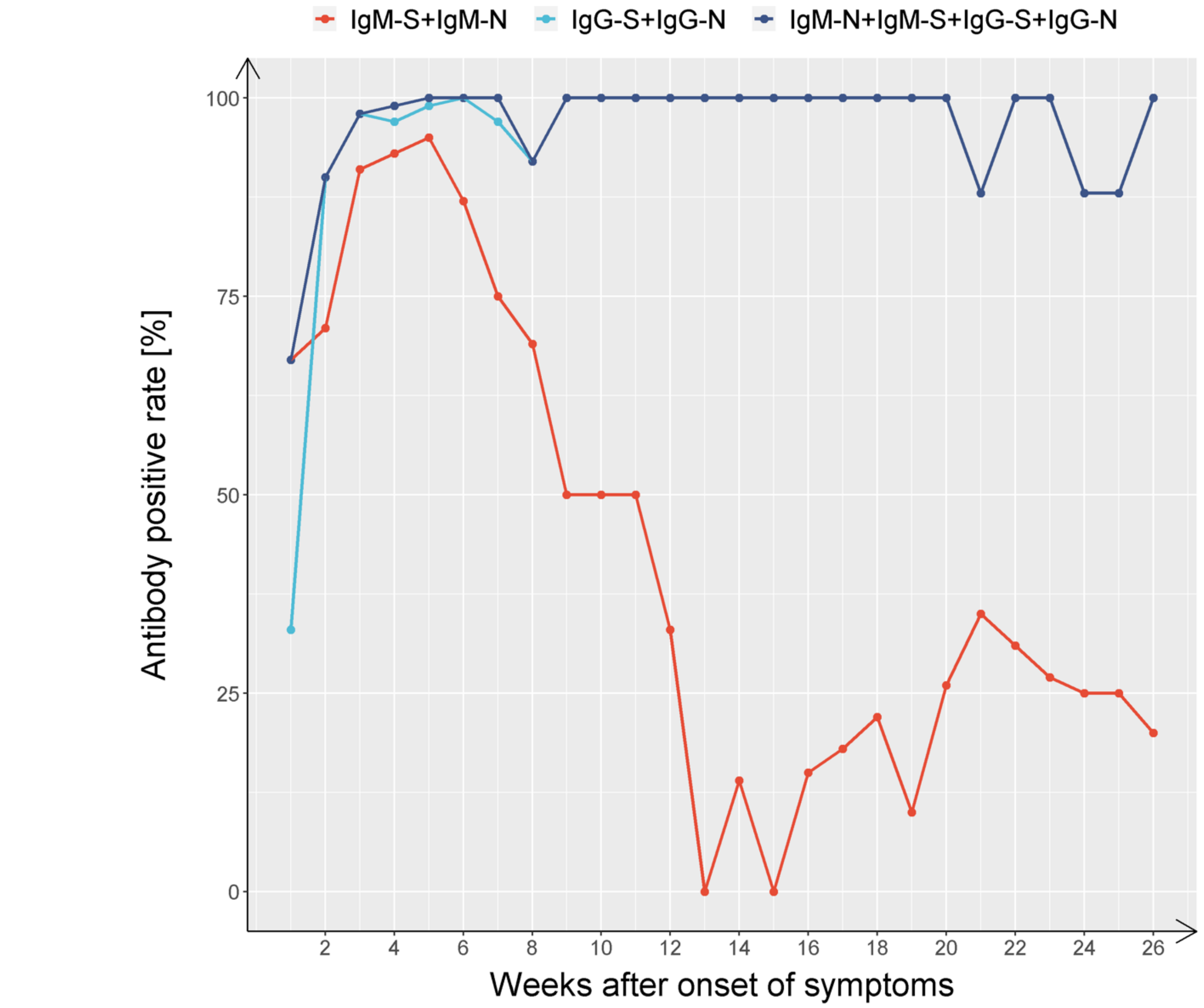

IgM-S+IgM-N [%]	67	71	91	93	95	87	75	69	50	50	50	33	0	14	0	15	18	22	10	26	35	31	27	25	25	20
IgG-S+IgG-N [%]	33	90	98	97	99	100	97	92	100	100	100	100	100	100	100	100	100	100	100	100	88	100	100	88	88	100
IgM-N+IgM-S+IgG-S+IgG-N [%]	67	90	98	99	100	100	100	92	100	100	100	100	100	100	100	100	100	100	100	100	88	100	100	88	88	100
Samples No.	9	58	43	69	79	61	32	13	2	4	6	9	9	22	10	20	22	23	10	19	17	16	11	8	8	5
Patients No.	9	56	41	64	68	54	31	13	2	4	6	9	9	22	10	20	22	23	10	19	17	16	11	8	8	5

Supplementary figure 1. Positive rate of combined antibodies following the onset of symptoms. IgM and IgG against the RBD of the spike protein and nucleoproteins of SARS-CoV-2 were detected by capture chemiluminescence immunoassays (CLIA). Positive rate of IgM-S + IgM-N, IgG-S + IgG-N, IgM-S + IgM-N + IgG-S + IgG-N tested in time series after the onset of symptoms.

a

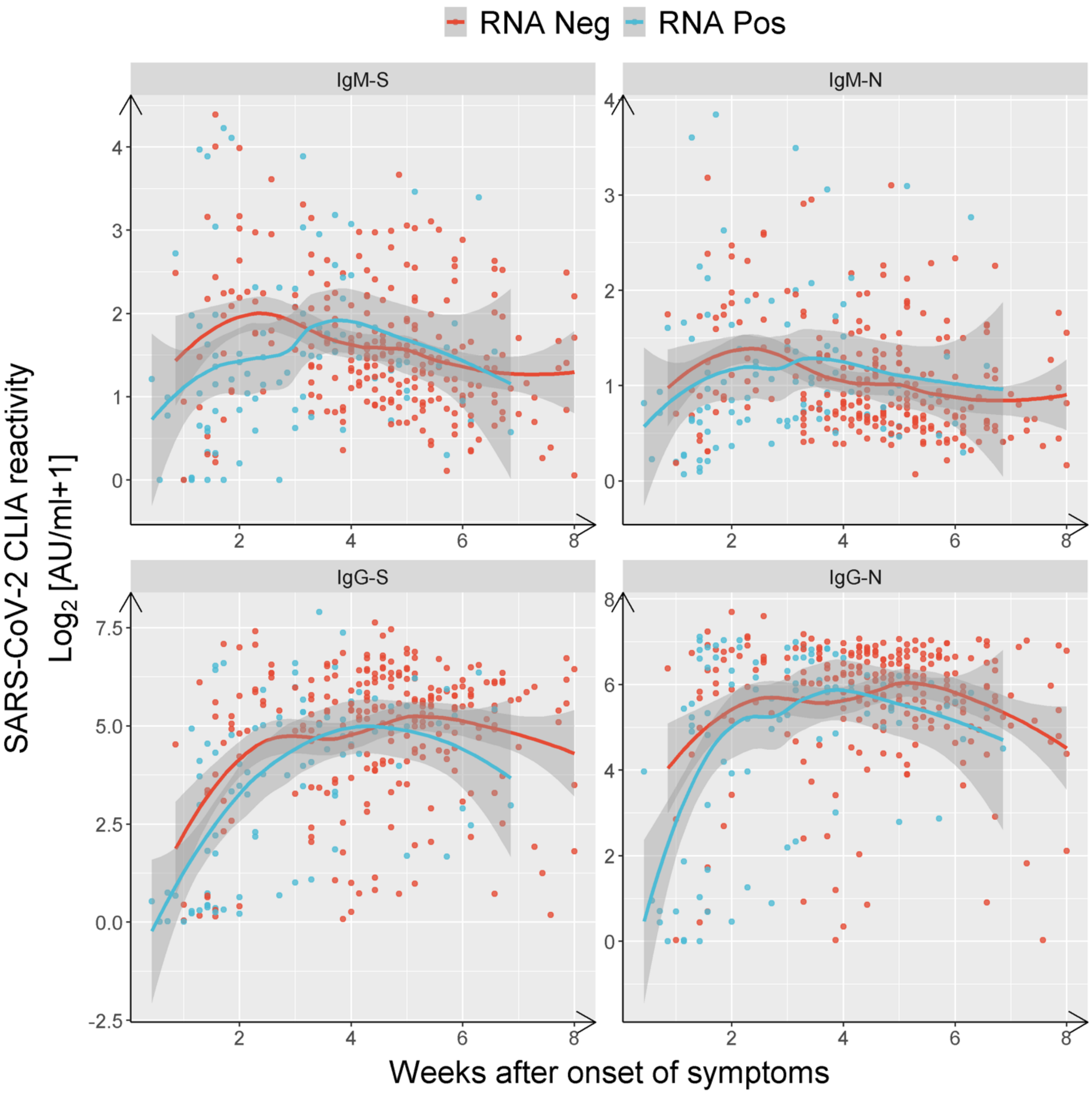
RNA Neg
RNA Pos
IgM-S
IgM-N
IgG-S
IgG-N
SARS-CoV-2 CLIA reactivity
Log$_2$ [AU/ml+1]
Weeks after onset of symptoms

b

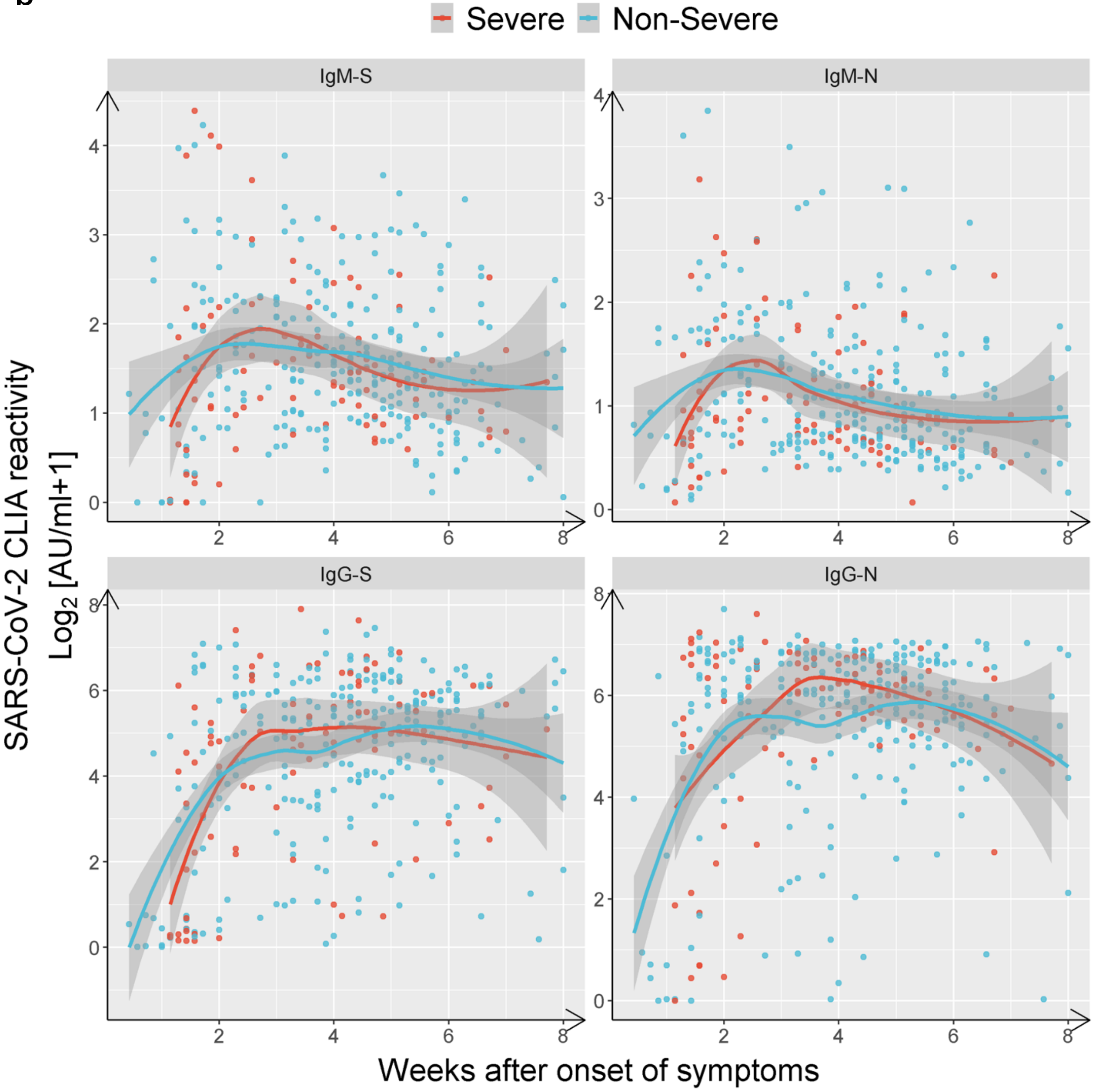
Severe
Non-Severe
IgM-S
IgM-N
IgG-S
IgG-N
SARS-CoV-2 CLIA reactivity
Log$_2$ [AU/ml+1]
Weeks after onset of symptoms

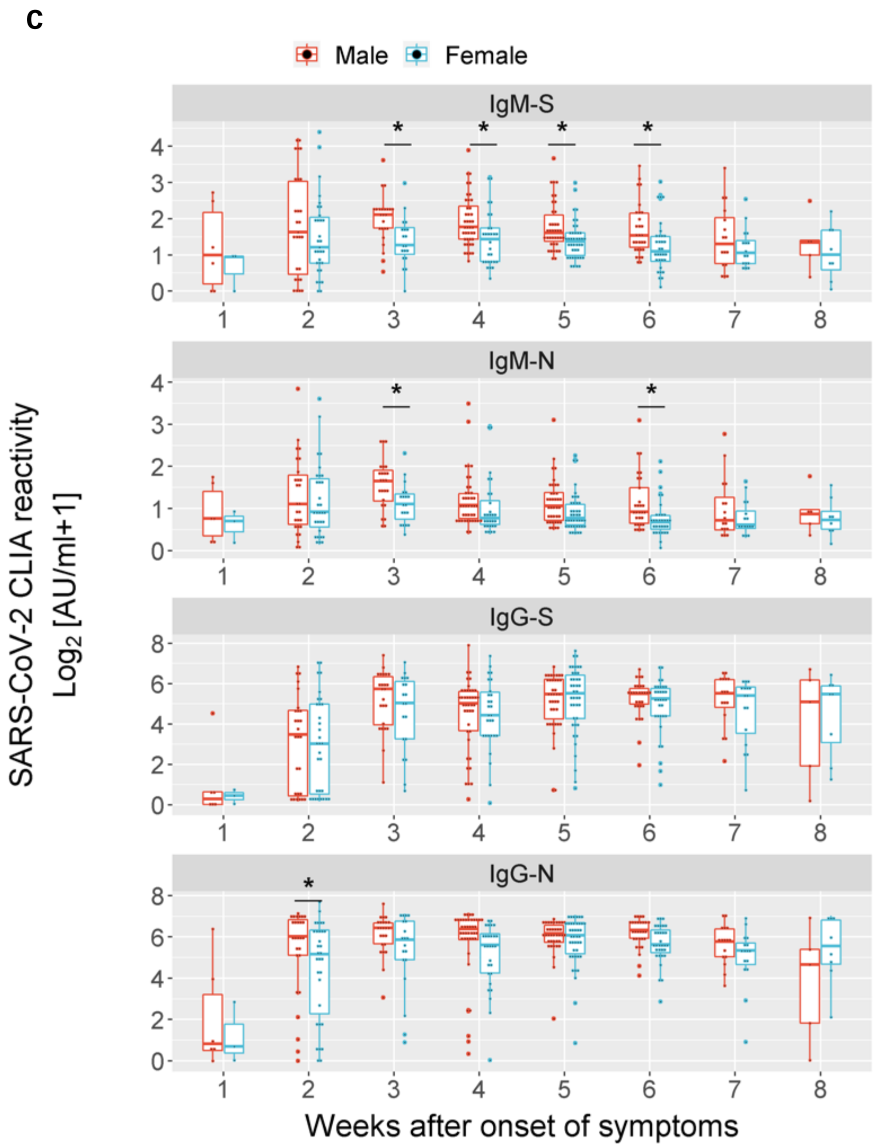

**Male [No.]**	6	27	24	41	38	29	16	5
**Female [No.]**	3	31	19	28	41	32	16	8

d

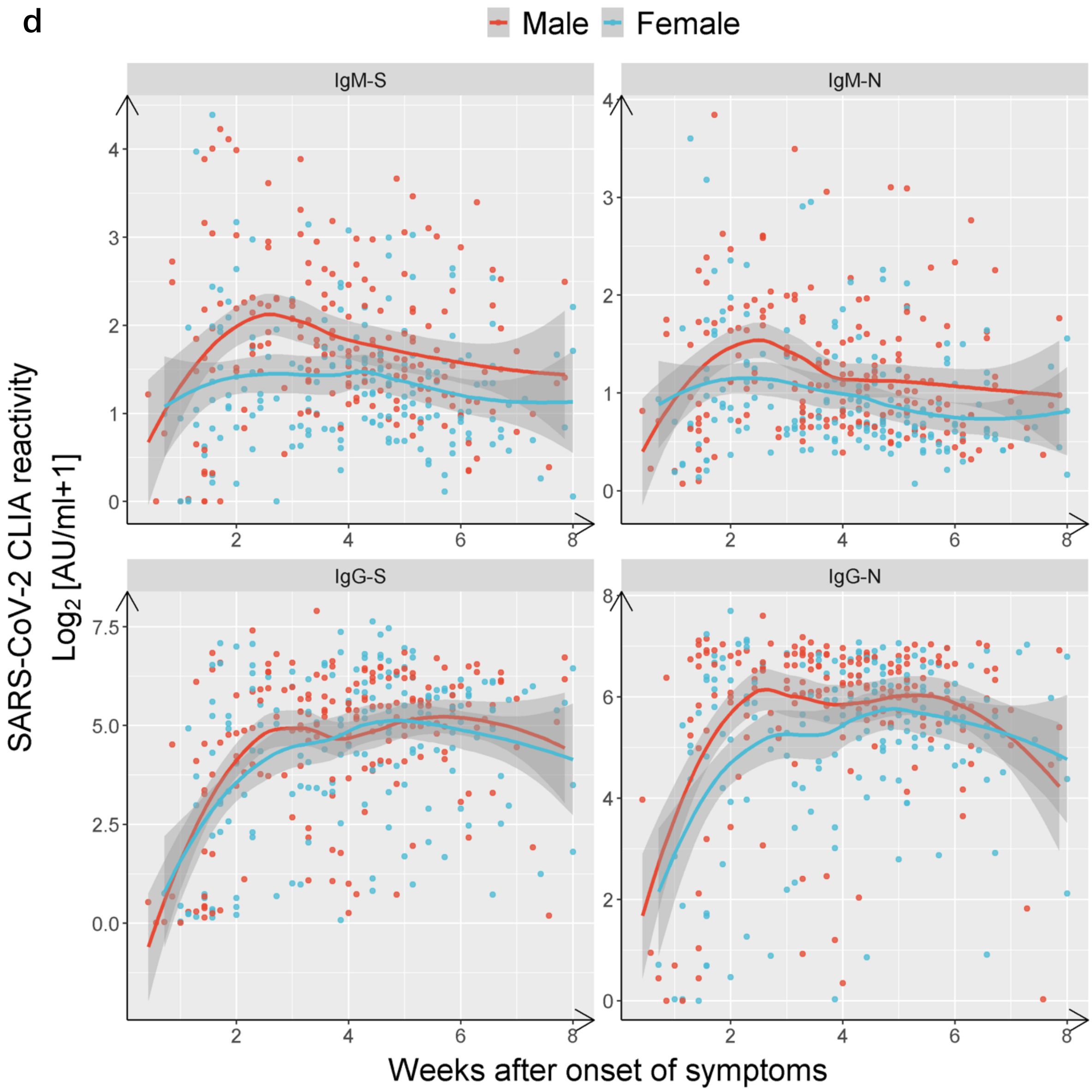
Male
Female
IgM-S
IgM-N
IgG-S
IgG-N
SARS-CoV-2 CLIA reactivity
Log2 [AU/ml+1]
Weeks after onset of symptoms

18

19

e

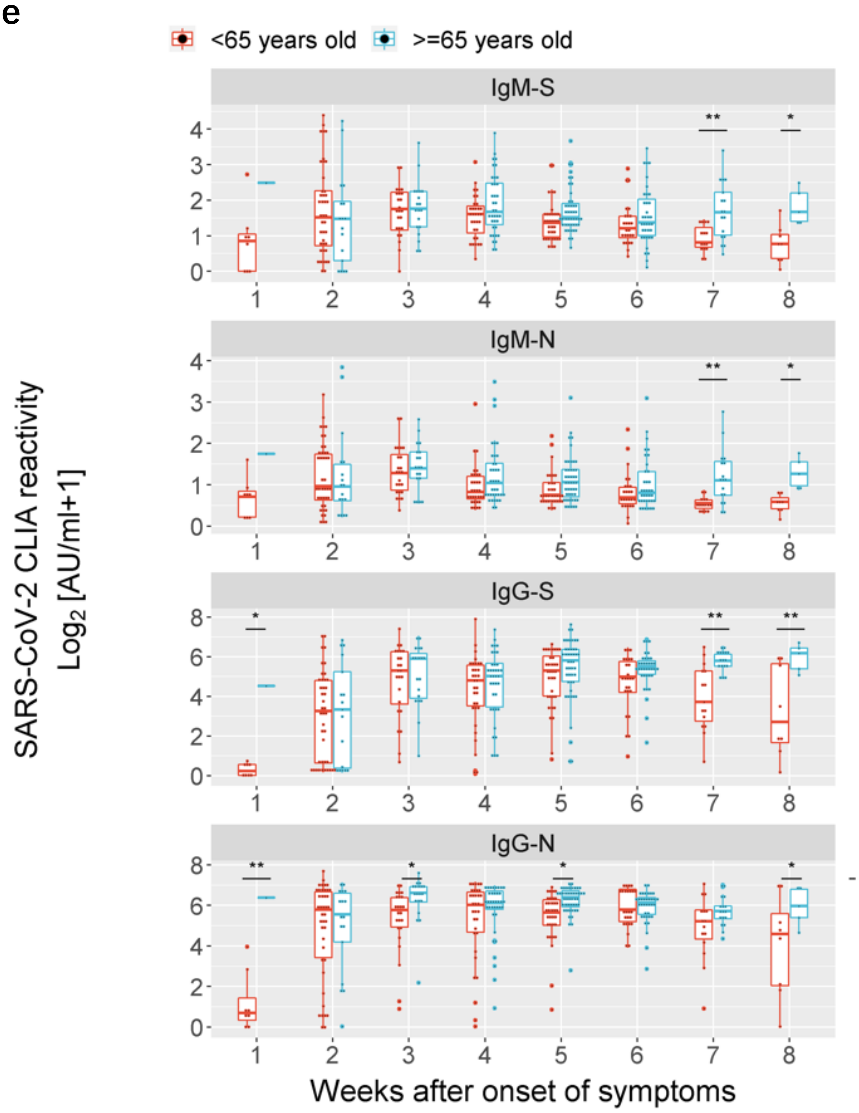

**<65 years old [No.]**	8	41	24	32	34	25	15	8
**>=65 years old [No.]**	1	17	19	37	45	36	17	5

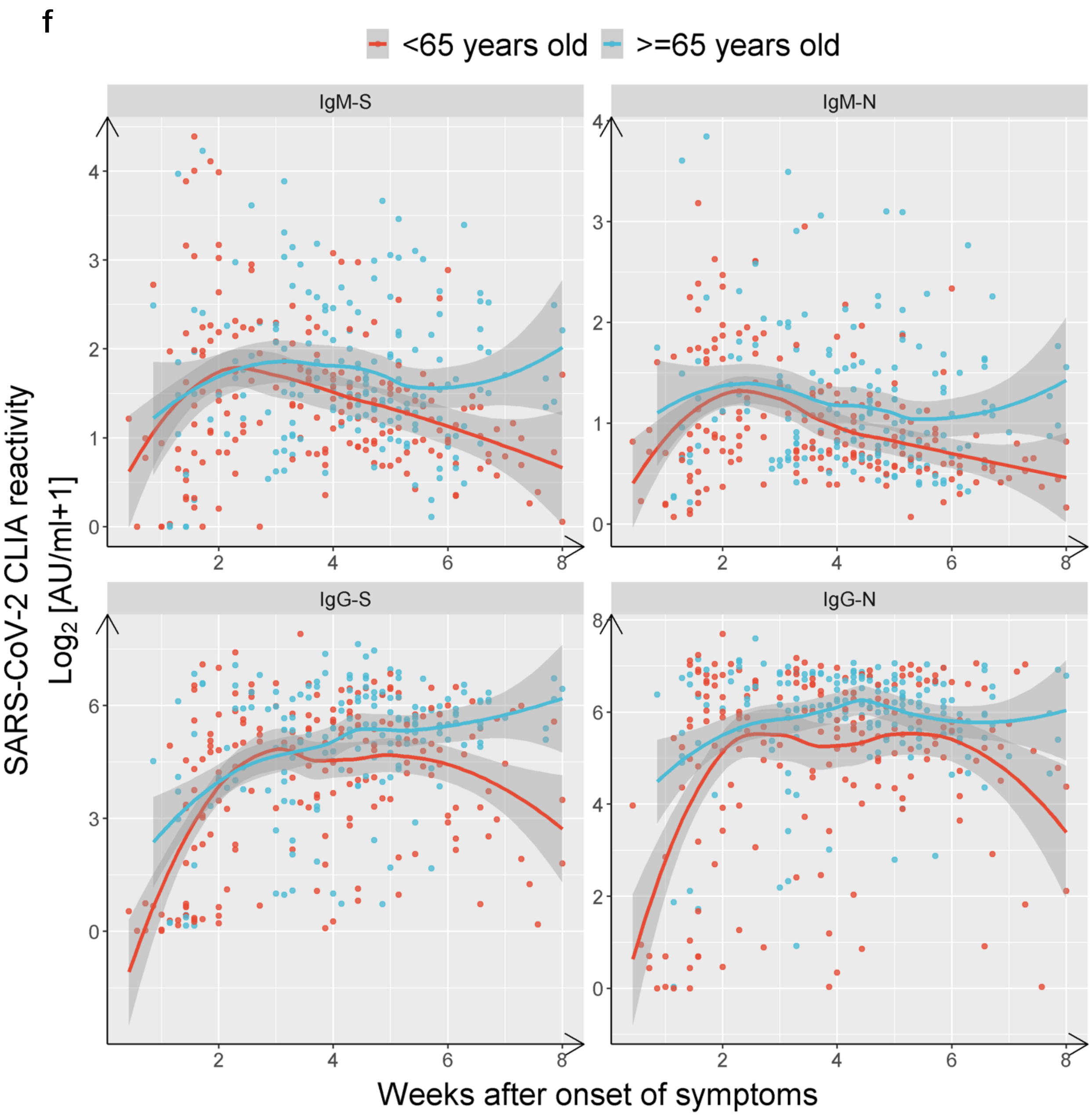
f
<65 years old
>=65 years old
IgM-S
IgM-N
IgG-S
IgG-N
SARS-CoV-2 CLIA reactivity
Log2 [AU/ml+1]
Weeks after onset of symptoms

Supplementary figure 2. Comparison of differences in antibody titers according to severity of disease, gender, and age. Comparison of S and N-specific CLIA-reactive IgM/IgG titers between RNA-positive and -negative cases **(a)**, severe and non-severe patients **(b),** female and male patients **(c-d)** and the young (<65 years old) and elder (≥65 years old) patients (**e-f).** The lines in Fig S2a, S2b, S2d and S2f show the mean values expected from a Lowess regression model, with shaded area representing 95% confidence interval, and each blot represents one sample in this analysis. The table below the figure represents the number of samples at each time point. The boxes in Fig S2c and S2e show medians (middle line), 75% quartiles (upper bound) and 25% quartiles (lower bound), and the whiskers show 1.5× the IQR above and below the box. Repeated measures (mixed model) ANOVA was used for statistical analysis. *p<0.05; **p <0.01; ***p<0.001, two-sided. Adjusted P values for comparison of S and N-specific CLIA-reactive IgM/IgG titers between female and male patients are as follows: 2nd week after onset: IgG-N (P=0.031); 3rd week after onset: IgM-S (P=0.013); IgM-N (P=0.019); 4th week after onset: IgM-S (P=0.013); 5th week after onset: IgM-S (P=0.042); 6th week after onset: IgM-S (P=0.030); IgM-N (P=0.019); 7th week after onset: IgM-N (P=0.023);8th week after onset: IgM-N (P=0.023). Adjusted P values for comparison of S and N-specific CLIA-reactive IgM/IgG titers between the young (<65 years old) and elder (≥65 years old) patients are as follows: 1st week after onset: IgG-S（P=0.019）；IgG-N（P=0.002）; 3rd week after onset: IgG-N（P=0.033）; 5th week after onset: IgG-N (P=0.032); 7th week after onset: IgM-S (P=0.008)；IgM-N (P=0.004)；IgG-S (P=0.002)；8th week after onset: IgM-S (P=0.023)；IgM-N (P=0.038)；IgG-S (P=0.005)；IgG-N (P=0.028).

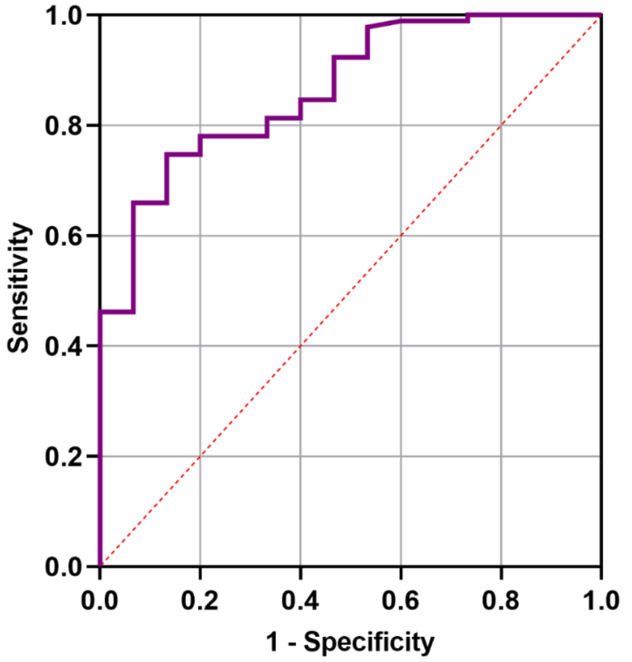

Variable	AUC	95%CI	P value	Cut-off
**IgG-S**	0.865	0.777-0.953	<0.0001	4.99

Supplementary figure 3. ROC curve of IgG-S titers to predict neutralizing activity of COVID-19 patients. The AUC of IgG-S was 0.865 (95% CI 0.777–0.953; $p<0.0001$). The optimal cutoff value was 4.99 AU/ml.

Supplementary table 1. Clinical and laboratory characteristics of hospitalized COVID-19 patients

Characteristic	Non-severe,（n=149）	Severe,（n=60）	*P* value
**Age [years]**	60.0 (43.5-68.0)	60.0 (47.3-67.8)	0.522[b]
**Female, sex [n %]**	76 (51.0%)	23 (38.3%)	0.125[a]
**WBC [10^9/L]**	5.2 (4.4-6.8)	5.5 (4.2-7.9)	0.199[b]
**N [10^9/L]**	3.3 (2.7-4.5)	4.4 (2.8-6.3)	**0.004**[b]
**L [10^9/L]**	1.4 (1.0-1.7)	0.9 (0.6-1.2)	**<0.001**[b]
**M [10^9/L]**	0.5 (0.4-0.7)	0.5 (0.3-0.6)	0.546[b]
**N [%]**	63.3 (55.4-70.9)	71.7 (59.1-79.9)	**0.001**[b]
**L [%]**	25.3 (19.2-33.7)	20.1 (11.8-31.1)	**0.005**[b]
**M [%]**	8.9 (6.9-11.0)	7.3 (5.2-9.3)	**0.001**[b]
**PLT [10^9/L]**	211.0 (170.5-275.0)	200.5 (143.8-236.8)	**0.029**[b]
**TBil [μmol /L]**	10.9 (9.0-14.3)	12.9 (9.7-16.5)	**0.021**[b]
**ALT [U/L]**	25.0 (18.0-45.0)	37.0 (24.0-54.0)	**0.007**[b]
**AST [U/L]**	24.0 (19.0-34.5)	40.0 (28.0-56.0)	**<0.001**[b]
**LDH [U/L]**	224.0(181.0-288.0)	355.0 (271.5-449.0)	**<0.001**[b]
**CK [U/L]**	62.0 (43.0-88.0)	76.0 (48.5-200.5)	**0.016**[b]
**Cr [μmol /L]**	67.4 (55.7-79.0)	72.0 (58.0-86.4)	**0.046**[b]
**D-Dimer [mg/L]**	0.5 (0.2-1.0)	0.9 (0.4-2.9)	**0.001**[b]
**PT [s]**	13.2 (12.7-13.8)	13.6 (12.9-14.4)	**0.030**[b]
**TT [s]**	17.6 (16.6-18.5)	17.5 (16.6-18.6)	0.785[b]
**APTT [s]**	37.0 (35.1-40.0)	37.7 (35.0-41.4)	0.436[b]
**FIB [g/L]**	4.2 (3.4-5.1)	5.4 (4.4-6.7)	**<0.001**[b]

WBC: white blood cells, N: neutrophil count, M: monocyte count, L: lymphocyte count, N%: neutrophil percentage, M%: monocyte percentage, L%: lymphocyte percentage, PLT: platelet, total bilirubin (TBil), ALT: alanine aminotransferase, AST: aspartate aminotransferase, CK: creatine kinase, LDH: lactate dehydrogenase, Cr: Creatinine and prothrombin time.

All data are presented as the median (IQR) or n (%).

All data are calculated applying a $\chi 2$ test (a) or two-tailed Mann-Whitney U test (b).

P <0.05 was considered statistically significant.

Supplementary table 2. Realtime RT-PCR primers and probes used for SARS-CoV-2 detection

Gene	Primer	Sequence (5' to 3')
N	Probe	FAM-TTGCCCCCAGCGCTTCA-BHQ1
	Forward	TTGGGGACCAGGAACTAAT
	Reverse	GAAGGTGTGACTTCCATGC
ORF1a/b	Probe	HEX-TCCCACCCAAGAATAGCATAGATGC-BHQ1
	Forward	TTTAGATATATGAATTCACAGGGA
	Reverse	ACCAACACCCAACAATTTAAT
RNP	Probe	Cy5-TCCACAAGTCCGCGCAGAG-BHQ2
	Forward	AGATTTGGACCTGCGAG
	Reverse	ACTGAATAGCCAAGGTGAG
