# Supplementary material for: SARS-CoV-2 infection induces sustained humoral immune responses in convalescent patients following symptomatic COVID-19
Source: Nat Commun. 2021 Mar 22;12:1813. doi: 10.1038/s41467-021-22034-1 (PMC7985370; doi:10.1038/s41467-021-22034-1)
Supplement: Supplementary file 3 — Reporting Summary [file 41467_2021_22034_MOESM3_ESM.pdf]

nature research

Corresponding author(s): Xin Zheng

Last updated by author(s): Jan 1, 2021

Reporting Summary

Nature Research wishes to improve the reproducibility of the work that we publish. This form provides structure for consistency and transparency in reporting. For further information on Nature Research policies, see our Editorial Policies and the Editorial Policy Checklist.

Statistics

For all statistical analyses, confirm that the following items are present in the figure legend, table legend, main text, or Methods section.

n/a	Confirmed	
☐	☒	The exact sample size (*n*) for each experimental group/condition, given as a discrete number and unit of measurement
☐	☒	A statement on whether measurements were taken from distinct samples or whether the same sample was measured repeatedly
☐	☒	The statistical test(s) used AND whether they are one- or two-sided<br>*Only common tests should be described solely by name; describe more complex techniques in the Methods section.*
☒	☐	A description of all covariates tested
☒	☐	A description of any assumptions or corrections, such as tests of normality and adjustment for multiple comparisons
☐	☒	A full description of the statistical parameters including central tendency (e.g. means) or other basic estimates (e.g. regression coefficient) AND variation (e.g. standard deviation) or associated estimates of uncertainty (e.g. confidence intervals)
☒	☐	For null hypothesis testing, the test statistic (e.g. *F*, *t*, *r*) with confidence intervals, effect sizes, degrees of freedom and *P* value noted<br>*Give P values as exact values whenever suitable.*
☒	☐	For Bayesian analysis, information on the choice of priors and Markov chain Monte Carlo settings
☒	☐	For hierarchical and complex designs, identification of the appropriate level for tests and full reporting of outcomes
☐	☒	Estimates of effect sizes (e.g. Cohen's *d*, Pearson's *r*), indicating how they were calculated

Our web collection on statistics for biologists contains articles on many of the points above.

Software and code

Policy information about availability of computer code

Data collection	No software was used.
Data analysis	All statistical analysis was conducted by R (The R Foundation, http://www.r-project.org, version 4.0.0; ggplot2 package:https://cran.r-project.org/web/packages/ggplot2/index.html) and SPSS (version 25, IBM, USA)

For manuscripts utilizing custom algorithms or software that are central to the research but not yet described in published literature, software must be made available to editors and reviewers. We strongly encourage code deposition in a community repository (e.g. GitHub). See the Nature Research guidelines for submitting code & software for further information.

Data

Policy information about availability of data

All manuscripts must include a data availability statement. This statement should provide the following information, where applicable:

- Accession codes, unique identifiers, or web links for publicly available datasets
- A list of figures that have associated raw data
- A description of any restrictions on data availability

We can provide participant data without names and identifiers for the purpose of protecting the patients' privacy. We deposited a 'Source Data' Excel file comprising the data of each figure/table in a separate sheet to a repository named 'Science Data Bank' (DOI: 10.11922/sciencedb.00312; CSTR: 31253.11.sciencedb.00312; PID: 21.86116.6/sciencedb.00312). Our data include patients' clinical test results (such as whole blood routine, liver and kidney function, coagulation function, etc.), SARS-Cov-2 antibody test results and neutralizing activity experiment results.

Field-specific reporting

Please select the one below that is the best fit for your research. If you are not sure, read the appropriate sections before making your selection.

- [x] Life sciences
- [ ] Behavioural & social sciences
- [ ] Ecological, evolutionary & environmental sciences

For a reference copy of the document with all sections, see nature.com/documents/nr-reporting-summary-flat.pdf

Life sciences study design

All studies must disclose on these points even when the disclosure is negative.

Sample size	Sample size was chosen based on availability of plasma samples collected at multiple time points from patients admitted to Union Hospital. By July 15th, 2020, 585 samples obtained from 349 symptomatic COVID-19 patients of the isolation wards, fever clinics of Wuhan Union Hospital or National Virus Resource Center of Wuhan Institute of Virology were involved in this study. This was considered sufficient for an observational study
Data exclusions	We first collected blood samples from all patients, but established exclusion criteria before analyzing the data. We excluded individuals who were co-infected with human influenza A virus, influenza B virus, or other viruses related to respiratory tract infections, because co-infection with other pathogens affects the patient's antibody level. We also excluded some samples from patients whose antibody levels were repeatedly tested within three days, which would reduce the bias caused by artificial repeated testing.
Replication	The Precision and reproducibility of the capture chemiluminescence immunoassays (CLIA) assays was conformed where the same serum samples (n=100) were run independently in two experiments and very similar results were obtained for the independent experiments. Serum samples used for detection of antibody titer were run once tested in our study. Reproducibility of neutralization activity were measured for a subset of serum samples (n=50) and very similar results were seen between the independent experiments. The neutralization activity was tested one time in triplicates for each serum sample in our study.
Randomization	This study is an observation study, not an intervention trial, so no randomization is needed here.
Blinding	Serum treatment and antibody detection were performed independently by technician blind to samples information, data analysis was performed by three trained researchers.

Reporting for specific materials, systems and methods

We require information from authors about some types of materials, experimental systems and methods used in many studies. Here, indicate whether each material, system or method listed is relevant to your study. If you are not sure if a list item applies to your research, read the appropriate section before selecting a response.

Materials & experimental systems

n/a	Involved in the study	
[ ]	[x]	Antibodies
[ ]	[x]	Eukaryotic cell lines
[x]	[ ]	Palaeontology and archaeology
[x]	[ ]	Animals and other organisms
[ ]	[x]	Human research participants
[x]	[ ]	Clinical data
[x]	[ ]	Dual use research of concern

Methods

n/a	Involved in the study	
[x]	[ ]	ChIP-seq
[x]	[ ]	Flow cytometry
[x]	[ ]	MRI-based neuroimaging

Antibodies

Antibodies used	The capture chemiluminescence immunoassays (CLIA) kit used to detect the antibodies levels contains anti-human IgM/IgG monoclonal antibody, but the supplier name, catalog/lot number and clone name are not notified in the kit manual.
Validation	*Describe the validation of each primary antibody for the species and application, noting any validation statements on the manufacturer's website, relevant citations, antibody profiles in online databases, or data provided in the manuscript.*

Eukaryotic cell lines

Policy information about cell lines

Cell line source(s)	Vero E6 cell line (ATCC; CRL-1586, Lot#: 60526234
Authentication	No authentication was performed.

Mycoplasma contamination	These cell lines were tested negative for mycoplasma.
Commonly misidentified lines (See ICLAC register)	No commonly misidentied cell lines were used.

Human research participants

Policy information about studies involving human research participants

Population characteristics	A total of 585 samples obtained from 349 symptomatic COVID-19 patients, collected up to 26 weeks after disease onset. 149 (71.3%) non-severe cases and 60 (28.7%) severe cases from isolation wards with complete medical records were enrolled. No significant dilerences concerning gender and age were observed between these two groups.
Recruitment	585 samples obtained from 349 symptomatic COVID-19 patients of the isolation wards, fever clinics of Wuhan Union Hospital or National Virus Resource Center of Wuhan Institute of Virology, during the period January 1st to July 15th, 2020, were involved in this study. All patients in this study were diagnosed and treated according to the Guidelines of the Diagnosis and Treatment of New Coronavirus Pneumonia (version 7) published by the National Health Committee of the People's Republic of China.
Ethics oversight	This study was approved by the Ethics Commission of Union Hospital of Huazhong University of Science and Technology in Wuhan.

Note that full information on the approval of the study protocol must also be provided in the manuscript.
